# High 10-Year Survival Rate with an Anatomic Cementless Stem (SPS)

**DOI:** 10.1007/s11999-012-2300-0

**Published:** 2012-03-09

**Authors:** Elhadi Sariali, Alexandre Mouttet, Philippe Mordasini, Yves Catonné

**Affiliations:** 1 Department of Orthopaedic Surgery, Hôpital Pitié Salpétrière, 47-83 Bd de l’Hôpital, 75013 Paris, France; 2 Department of Orthopaedic Surgery, Clinique Saint Roch, Perpignan, France; 3 Department of Orthopaedic Surgery, Clinique Bois-Cerf, Lausanne, Switzerland

## Abstract

**Background:**

Proximal cementless fixation using anatomic stems reportedly increases femoral fit and avoids stress-shielding. However, thigh pain was reported with the early stem designs. Therefore, a new anatomic cementless stem design was based on an average three-dimensional metaphyseal femoral shape. However, it is unclear whether this stem reduces the incidence of thigh pain.

**Questions/purposes:**

We asked whether this stem design was associated with a low incidence of thigh pain and provided durable fixation and high function.

**Methods:**

One hundred seventy-one patients (176 THAs) who had the anatomic proximal hydroxyapatite-coated stem implanted were reviewed. Eleven (6%) patients were lost to followup and 34 (20%) died without revision surgery. We used the Harris hip score (HHS) to assess pain and function. We evaluated femoral stem fixation and stability with the score of Engh et al. and also calculated a 10-year survival analysis. We assessed 126 patients (131 hips) at a mean followup of 10 years (range, 8–11 years)

**Results:**

At last followup, two patients described slight thigh pain that did not limit their physical activities. All stems appeared radiographically stable and one stem was graded nonintegrated but stable. Five patients had revision surgery: one on the femoral side (for posttraumatic fracture) and four on the acetabular side. Considering stem revision for aseptic loosening as the end point, survivorship was 100% (range, 95.4%–99.9%) at 10 years.

**Conclusion:**

This anatomic cementless design using only metaphyseal fixation with a wide mediolateral flare, a sagittal curvature, and torsion, allowed durable proximal stem stability and fixation.

**Level of Evidence:**

Level IV, therapeutic study. See Guidelines for Authors for a complete description of levels of evidence.

## Introduction

The survivorship rates for uncemented stems are as good as those for cemented stems, but depend on the stem’s design, material, and type of coating [4, 6, 7]. Primary stability of the stem is crucial to insure bone ingrowth. Surgeons can achieve initial stability by filling the femoral diaphysis or getting intimate contact between the stem and the anatomy of the proximal femur. Many designs of proximally fixed cementless femoral stems have been developed to achieve physiologic bone remodeling [8, 14]. The designers assumed the implant would achieve primary stability and minimize stress shielding or overload.

Using a proximal metaphyseal fit cementless custom anatomic stem in patients younger than 50 years, Flecher et al. [14] reported high functional scores and a 15-year survival rate of 93% with minimal osteolysis, even in patients with severe deformities. These data suggested anatomic cementless stems with metaphyseal fit and fill can provide durable pain relief and function in physically active patients.

**Fig. 1:**
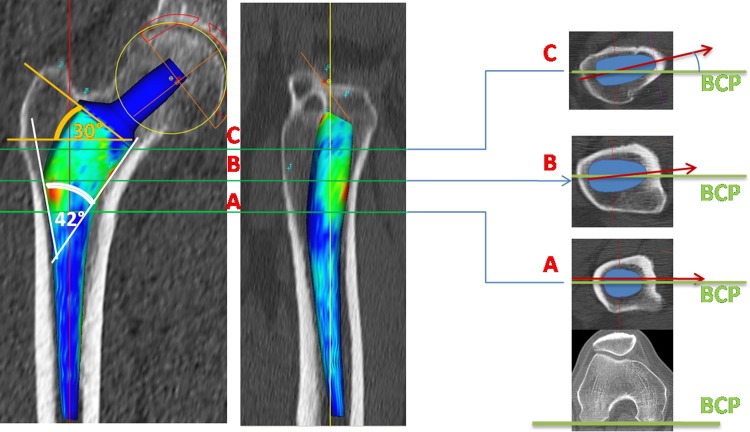
The SPS stem was designed to achieve intimate contact with the proximal femur. Fifteen degrees of anterior torsion of the upper portion of the stem was used to fit the natural femoral helitorsion. The axial cuts A, B, and C show the progressive helitorsion of the stem and the femur. The bicondylar plane of the knee (BCP) is used as a reference for the torsion. The stem design includes a wide mediolateral flare (42°) which permits a horizontal neck osteotomy (30°) and therefore bone preservation.

Based on the experience of making custom prostheses [13], a cementless anatomic stem was designed using a computer-assisted technique to achieve intimate contact between the stem and the anatomic shape of the proximal femur. The design was based on a database of 300 hip CT images to approximate an average intracanalar shape of the proximal femoral metaphysis and obtain reference values for torsion of the proximal femur in the axial and sagittal planes [23]. The stem was designed with an anterior torsion of 15° and an anterior sagittal curvature to fit the average proximal femur (Fig. 1), presuming this shape would ensure good primary stability and long-term fixation. In an earlier study [28], a 5-year overall survival rate of 98.8% was reported for 171 patients (176 hips). To confirm those findings, we now report on the same cohort at longer followup.

We therefore asked whether these anatomic cementless stems provided (1) a low incidence of thigh pain, (2) stable fixation, (3) high long-term survivorship, (4) high functional scores, and (5) a low rate of wear.

## Patients and Methods

**Fig. 2:**
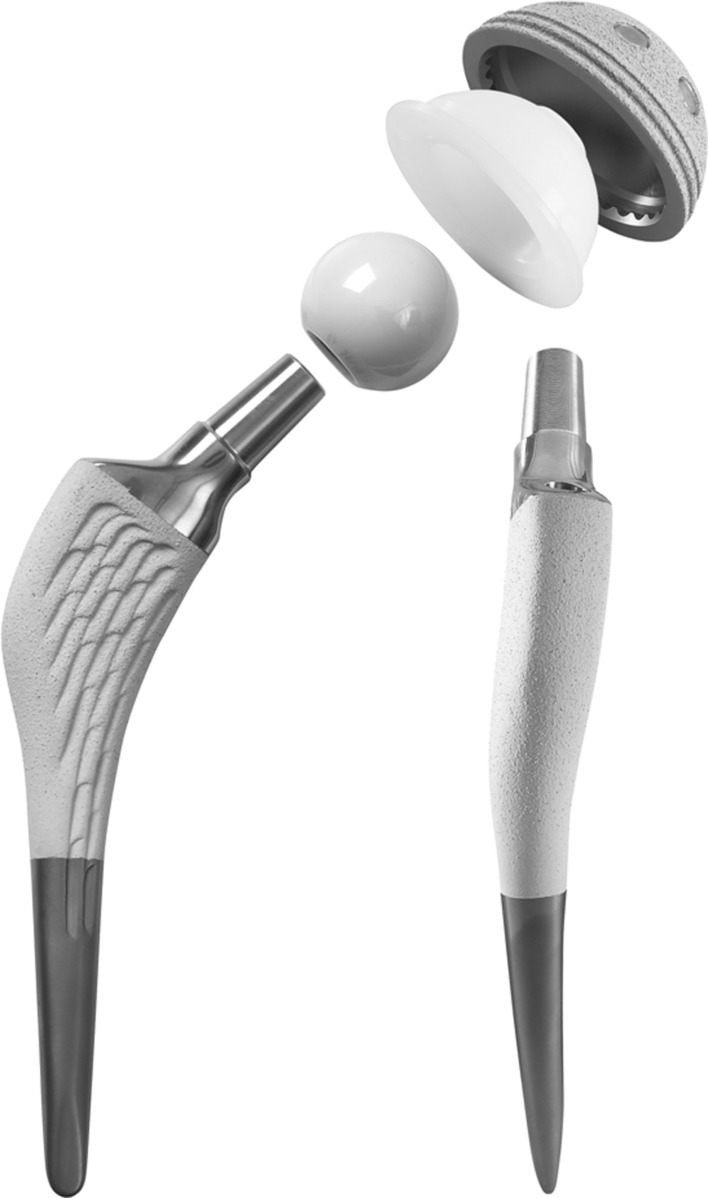
The anatomic cementless SPS stem is shown.

We retrospectively reviewed all 171 patients (176 hips) who underwent THA between September 1, 1997 and December 31, 1998, using the SPS anatomic proximally hydroxyapatite (HA)-coated stem (Symbios, Yverdon-les-Bains, Switzerland) (Fig. 2) and an HA-coated acetabular component with zirconia-ceramic and UHMWPE liner (Symbios). During the study period all patients having THA were treated with the same implants. The indications for these particular implants were: (1) primary osteoarthritis, (2) developmental dysplasia of the hip (DDH), avascular necrosis, (3) inflammatory arthritis, or (3) posttraumatic osteoarthritis. The contraindications were: (1) revision THA, or (2) hip fractures. There were 102 women and 69 men, with a mean age of 73 years (range, 35–83 years) at the time of surgery and a mean BMI of 26 kg/m^2^ (range, 17–35 kg/m^2^); 83 (49%) patients were overweight and 25 (15%) were obese. Diagnoses included primary osteoarthritis (132 [75%]), DDH (18 [10%]), osteonecrosis (14 [8%]), inflammatory arthritis (10 [6%]), or posttraumatic osteoarthritis (two [1%]). Eleven patients (6%) were lost to followup and 34 (20%) died without any revision surgery performed. This left 126 patients (131 hips) for review. The minimum followup was 8 years (mean, 10 years; range, 8–11 years).

One surgeon (AM) performed all operations using an anterolateral approach with the patient in the supine position. The femoral stem had a lateral flare designed to ensure primary craniocaudal stabilization. Thus, we always prepared the proximal femoral canal with a curette before rasping. First we removed cancellous bone in contact with the lateral cortex under the greater trochanter to prepare the place where the lateral flare of the stem was located, and second, we removed any excessive femoral “internal calcar septum” [10] (the internal vertical plate of condensed trabecular bone constituting the anatomic calcar).

Postoperative rehabilitation protocols included immediate weightbearing protected by crutches during the first 2 or 3 weeks according to patient tolerance. The physiotherapy was supervised and was performed each day for at least 1 month. The exercises focused on passive and then active recuperation of ROM. All patients received routine thromboprophylaxis with low molecular-weight heparin postoperatively for 21 days.

**Fig. 3A–D:**
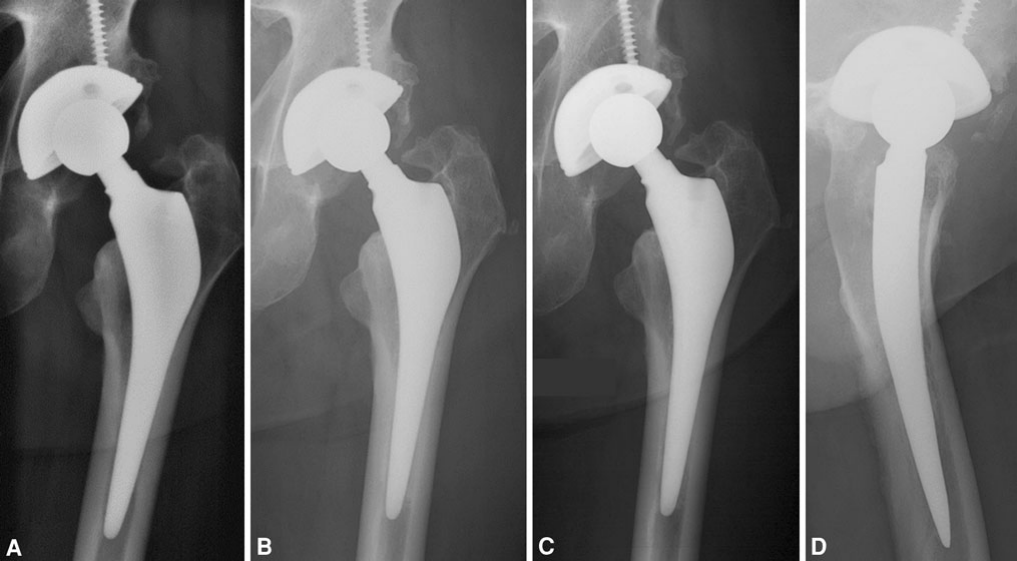
The implanted SPS stem had stable fixation at (**A**) 1, (**B**) 5, and (**C**) 10 years followup. (**D**) A lateral view obtained at the 10-year followup is shown.

Patients underwent evaluation at 3 months postoperatively, and then yearly until the last followup. One orthopaedic surgeon not involved in the treatment (PM) performed the last radiographic reviews (Fig. 3). We performed clinical evaluation using the HHS [18]. We recorded the presence or absence of thigh pain at each visit.

We performed radiographic postoperative evaluations using AP views of the pelvis and hip and a true lateral view of the hip. We looked for radiolucencies and osteolysis in the seven zones described by Gruen et al. [16] and the corresponding seven zones on the lateral view. Radiolucencies (progressive and greater than 2 mm) and osteolysis (defined as areas of reduced bone density not present on the initial radiographs) were recorded [9]. We evaluated the femoral component fixation and stability using the score described by Engh et al. [12], which included fixation score and stability scores. The fixation scale analyzed the appearance of the stem porous interface (reactive lines and lucencies) and the presence of spot welds. The absence of lines or lucencies and the presence of spot welds corresponded to bone ingrowth and were counted positively (+5 points) with a maximal score of 10. The stability scale was determined by comparing the postoperative and the last radiographs. We recorded the appearance of the smooth interface of the stem (lines or lucencies), presence of a pedestal with an unstable stem tip, calcar remodeling, deterioration of the integrated interface, stem migration, and presence of particle shedding. Positive scores were recorded if there were no lines or lucencies, no pedestal, no calcar hypertrophy, no interface deterioration, no migration, and no particle shedding. The maximal score using the criteria of Engh et al. was 27. Greater than 10, stem osteointegration was confirmed; from 0 to 10 points osteointegration was probable; between −10 to 0 the stem was considered nonintegrated but stable; and less than −10 points the stem was considered unstable. For the socket, we recorded radiolucencies and osteolysis in Zones 1 to 3 according to DeLee and Charnley [11]. The loosening criteria for the cup were those defined by Hodgkinson et al. [21]. Polyethylene wear was measured using Imagika^TM^ software (View Tech®, CMC Corp, Edison, NJ, USA). We used three points to measure the head diameter and three others for the cup, and then measured linear wear by calculating the distance from the center of the head to the center of the cup. An accuracy of 0.28 mm was reported for this technique when using a 28-mm head, a metal-back, and a design where the centers of the cup and the head coincided [15]. The cup abduction angle was measured on the AP view of the pelvis using the Imagika^TM^ software. This angle was defined with respect to the landmarks of the inferior aspect of the obturator foramen and the long axis of the projected ellipse of the face of the cup [30].

We performed a 10-year survival analysis using the Kaplan-Meier technique (with 95% CI). A multivariate analysis was performed to analyze the wear considering the following parameters: age, sex, BMI, activity level (HHS), and cup abduction angle.

## Results

At the last followup, two (1.6%) patients described occasional thigh pain not limiting their physical activities or requiring medication.

We considered all stems stable and integrated and one stem as nonintegrated but stable. On the femoral side, no patient had radiolucencies greater than 2 mm wide. We noted a cortical pedestal without pain in one patient. The mean total score of Engh et al. was 20 ± 5, with a mean fixation score of 8 ± 4 and mean stability score of 12 ± 4. On the acetabular side, osteolysis zones were observed in 11 hips.

**Fig. 4:**
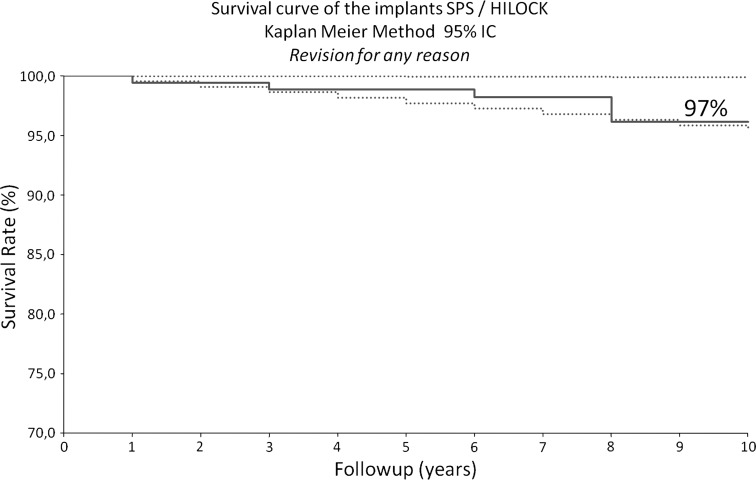
The graph shows the survival rate was 96.8% (95% CI), taking revision for any reason as an end point.

**Fig. 5:**
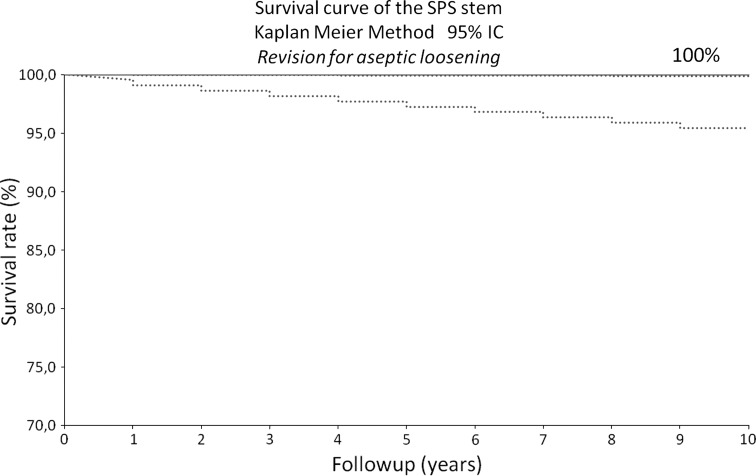
The graph shows the survival rate was 100% (95% CI) when taking revision for aseptic loosening of the stem as an end point.

Considering revision for any reason as the end point, mean survivorship was 97% (range, 95%–99.9%) at 10 years (Fig. 4). For stem revision for aseptic loosening as the end point, the survivorship was 100% (range, 95%–99.9%) at 10 years (Fig. 5), and for cup revision for aseptic loosening as the end point, the survivorship was 99.3% (range, 95%–99.9%) at 10 years. At last followup, five patients had undergone revision surgery: four patients on the acetabular side and one on the femoral side. We performed revisions for cup aseptic loosening in one patient (at 8 years), late instability related to severe wear in three patients (at 2, 8, and 8 years), and a posttraumatic femur fracture in one patient (at 2 years) (Table 1). No patient underwent revision surgery for femoral stem loosening.

**Table 1:** Details for patients who had revision surgery

Variables	Patient 1	Patient 2	Patient 3	Patient 4	Patient 5
Initial etiology	Primary osteoarthritis	Acetabular fracture	Primary osteoarthritis	Dysplasia	Dysplasia
Followup (years)	2	2	8	8	8
Cause for revision	Posttraumatic femur fracture	Recurrent anterior dislocation	Delayed recurrent dislocation	Cup aseptic loosening	Delayed recurrent dislocation
Osteolysis	No	No	Yes	No	No

The mean preoperative HHS improved at final followup from 37 to 90 (Table 2). Among the 126 remaining hips, 115 (92%) had no pain, 60 (48%) had full activity recovery, and 125 (99%) had full ROM.

**Table 2:** Comparison of preoperative and 10-year followup Harris hip scores

Harris hip score parameter	Preoperative Mean, SD, (range)	10-year followup Mean, SD, (range)	p value
Pain	9 ± 10 (0–30)	43 ± 4.8 (10–44)	< 0.0001
Walking	17 ± 8 (0–33)	27 ± 8 (2–33)	< 0.0001
Activity	6 ± 2 (0–12)	12 ± 3 (3–14)	< 0.0001
Deformity	1 ± 2 (0–4)	4 ± 0.3 (0–4)	< 0.0001
Range of motion	4 ± 1 (2–5)	4.9 ± 0.2 (4–5)	< 0.0001
Total	37 ± 17 (4–67)	90 ± 13 (45–100)	< 0.0001

The average linear wear was 0.09 mm per year (range, 0–0.26 mm per year).

Five patients had an early dislocation that did not require revision. We observed no other complications for this cohort and no zirconia head fractures were observed.

## Discussion

Proximal cementless fixation using anatomic stems is an attractive option for THA. However, an incidence of thigh pain of 5.7% to 17% was reported with the early designs [25, 35, 39] contrasting with the absence of pain reported by Flecher et al. [13, 14] with the anatomic custom stems. Therefore an anatomic cementless stem was designed, based on a computerized three-dimensional (3-D) analysis of a 300 CT scan database, which allowed defining an average metaphyseal femoral shape. We presumed this shape would provide a lower incidence of thigh pain than the previous designs, long-term stability and fixation, high 10-year Kaplan-Meier survivorship, high functional scores, and low rate of wear.

There were limitations to our study. First, we analyzed a small cohort with only 131 patients with a relatively older age at the time of surgery. The analysis of younger patients with high functional demand may be interesting because the mechanical constraints were expected to be higher. Second, we did not assess bone remodeling. A proper stem design allowing proximal fixation theoretically might help achieve more physiologic load transmission, enhancing more physiologic bone remodeling. A comparative study using DXA might assess whether the SPS stem effectively enhanced bone remodeling. Third, there was no control group.

High fixation and stability scores were achieved at 10 years followup with the SPS stem, which does not use diaphyseal anchorage. These findings compared well with those reported in the literature (Table 3), including studies analyzing stems with diaphyseal fixation. However, a direct comparison was difficult as few studies used the Engh scores. However, the lack of stability and fixation could be assessed clinically with the presence of thigh pain. Despite high survival rates, thigh pain has varied from 0% to 17% in patients [1, 25, 35, 39]. Many factors contributed to the long-term fixation and stability of the SPS stem. First, primary stability of the femoral component was ensured by intimate contact with the proximal femur, which was achieved by the anatomic design and axial torsion of the stem. Two other characteristics of the design also increased its stability: the wide mediolateral flare and the AP width (Fig. 1). The large flare (42°) of the SPS stem allowed it to be properly seated on the lateral and medial flares, minimizing the risk of frontal migration and stress shielding. As opposed to straight stems, the SPS component had a relatively greater AP width, improving its rotational stability. The sagittal curve of the stem allowed the use of a relatively wide width without generating difficulties during rasping and stem implantation. Furthermore, the anatomic design permitted a more horizontal neck osteotomy (approximately 30° inclination), preserving bone and increasing rotational stability. The close fit to the proximal femur allowed durable proximal stem fixation and more physiologic bone remodeling, suggesting that the SPS stem was a conservative implant. However, we postulated that the distal part of the stem may have been useful at the time of surgery to guide stem implantation and avoid frontal misalignment, although we did not shorten the stem despite that the stability and fixation were insured by the metaphyseal portion.

**Table 3:** Comparison of long-term followups of cementless polyethylene-bearing stems

Study	Stem	Number of hips	Mean followup (years)	Survival rate for aseptic loosening	Mean HHS	Thigh pain (%)	Stress shielding (%)	Bone ingrowth (%)	Wear (mm/year)
Aldinger et al. [1]	Sportono	354	12	98%	84	0	0%	99.7%	–
Baker et al. [3]	ABGI	69	15	100%	–	–	–	–	0.14
Flecher et al. [13]	Anatomic custom	233	15	97.6%	96.7	0	0%	–	0.09
Hennessy et al. [19]	Prodigy	82	11.4	100%	86	2	28%	100%	–
Kim et al. [25]	Profile	118	9.8	100%	92	10	–	98%	0.12
Lee et al. [27]	Omnifit	103	10.3	100%	92	0	0%	100%	0.24
Parvizi et al. [32]	Taperloc	129	11	99.1%	92	3.6	0%	100%	–
Schramm et al. [35]	CLS	89	10	100%	98	17	–	95%	–
Suckel et al. [37]	Zweymuller	320	17	98%	88	–	18%	–	–
Zenz et al. [39]	Zweymuller	56	10	99.3%	91	5.7	47%	–	–
Current study	SPS	176	10	100%	90	1.6	0%	99.3%	0.09

We achieved a high survival rate for the SPS stem at 10 years with high hip scores and no recorded loose stems. These findings compared well with those reported in the literature, especially when compared with the anatomic cementless stems [3, 5]. Other studies also reported high survival rates of proximally HA-coated, press-fit stems for different designs [19, 27, 32, 37]. Hennessy et al. [19] reported a 100% survival rate at 10 years with an extended, porous-coated, straight-stem with diaphyseal fixation (AML, DePuy, Warsaw, IN, USA). However, 28% of their patients had stress shielding and 2% reported thigh pain despite bone ingrowth being achieved by all patients. Parvizi et al. [32] reported no stem failures for the HA-coated Taperloc (Biomet, Warsaw, IN, USA) at 10 years followup. Lee et al. [27] also reported no stem failures with the quadrangular proximally coated stem Omnifit (Stryker, Mahwah, NJ, USA). Suckel et al. [37], using the Zweymüller stem (Alloclassic, Sulzer Orthopedics, Zimmer, Switzerland), found a similar survival rate of 98% at 15 years followup. The SPS design achieved a similar long-term survival rate when compared with these designs. The comparison of bone remodeling for all these designs may be useful in determining which ones more closely restored physiologic load transmission. The survivorship rate for the cup was 99.3%, which was similar to that reported in the literature for cementless cups [29].

The rate of patients with thigh pain was low in our cohort, comparing well with the lower rates of thigh pain reported in the literature for uncemented stems, which, although not always reported in studies, varies from 0% to 17% [1, 25, 35, 39]. Naumann et al. [31] reported 29% of patients had thigh pain during the first months after surgery and 5.6% had pain remaining afterward, for the first generation of the Zweymüller stem. They showed that pain was associated with stress shielding. Kim et al. [25] reported 10% of patients had thigh pain with the Profile anatomic stem, suggesting a lack of stability and fixation. Flecher et al. [14], however, reported no thigh pain with the anatomic custom stems, although the patients were younger than 50 years and with high functional demand. These findings suggested that a proper fit between the stem and the proximal femur may avoid thigh pain.

We observed a wear rate of approximately 0.09 mm per year, lower than the reported linear wear rates with metal-on-polyethylene components [20]. The reported wear rate for zirconia-polyethylene bearing surfaces varied from 0.09 [14] to 0.5 mm per year [2]. Some authors [2, 22] reported high wear rates for zirconia-polyethylene. These authors linked these high wear rates to the zirconia transformation phase, which reportedly induced an increase in the surface roughness [2, 36]. However, in another study, low wear rates similar to ours were reported when using zirconia-polyethylene bearings [5]. The main reason for revision in our series (three of the four cup revisions) was for late instability related to acetabular wear. Therefore, we now use alumina ceramic-on-ceramic bearing surfaces in younger or high-function patients.

To our knowledge, no other stem design has been based on a computerized technique to analyze the 3-D anatomy of the intracanalar proximal femur. We believe the anatomic SPS stem allowed correct transmission of physiologic loading to the metaphyseal cancellous bone, minimizing proximal stress-shielding and enhancing bone remodeling around the stem in the proximal femur. Proximal stress shielding, which has been associated with bone resorption around stems [24], could induce implant loosening [38] and should be avoided. The stable long-term fixation we observed with the SPS stem has supported this assumption because we observed no aseptic loosening and proximal fixation was achieved. However, we did not analyze bone remodeling with DXA; therefore, we could not draw a valid conclusion regarding the ability of the design to restore physiologic load transmission. Chen et al. [8] used DXA to analyze bone remodeling after THA, and proved that metaphyseal fixation allowed correct bone remodeling around the femoral stem. This may have explained our low rate of thigh pain (1.6%), despite the fact that no diaphyseal fixation was achieved. Thigh pain has been reported in as much as 16% of patients when using anatomic uncemented stems [17].

**Fig. 6A–C:**
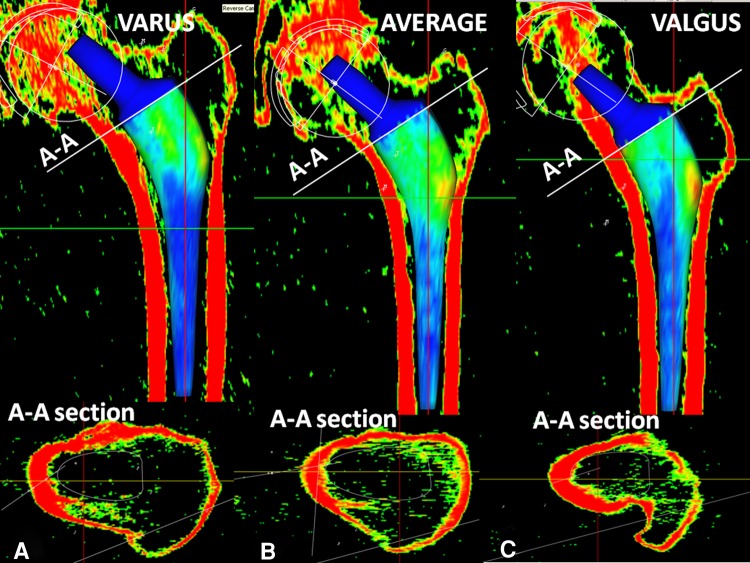
(**A**) With varus morphotypes the SPS stem may not have a good medial fit on the medial cortex. (**B**) The average SPS shape fits in almost all patients who do not have major dysplasia. (**C**) In case of a valgus morphotype, the SPS stem may have a punctual contact medially at the upper portion of the neck with a risk of fracture. A-A is the cross section corresponding to the neck osteotomy plane.

In contrast to the custom anatomic stems, the average shape of the SPS stem may not have a proper fit in some patients. These outliers may be detected before surgery by performing 3-D computerized planning [34]. In case of a varus morphotype, the stem may not have ideal medial mechanical support. However with a valgus morphotype, as the medial femoral cortex has a lower curvature, the medial contact with the stem may occur on a very small contact area located at the upper portion of the neck, generating higher mechanical constraints medially and therefore a risk of fracture (Fig. 6). In our experience, three types of proximal femur morphotypes exist (Fig. 6). The standard morphotype corresponds to the average intracanalar metaphyseal volume, which we used to design the proximal shape of the SPS stem. The valgus morphotype has a narrower medial flare, so the medial cortex has a lower curvature. However, the varus morphotype has a wider medial flare with a greater curvature of the medial cortex. Theoretically, three types of anatomic stems might be required to achieve intimate contact between the stem and the proximal femur, with a fit and fill equivalent to a custom anatomic stem. However, the high 10-year survival rate of the SPS stem suggests that this average shape seems appropriate for a majority of patients who do not have a major dysplasia. However, custom anatomic stems also allow more accurate reconstruction of the extramedullary anatomy, including the neck length, offset, and femoral anteversion. Some authors [26, 33] reported there is poor correlation between the intracanalar anatomy of the proximal femur and the extramedullary anatomy. Therefore, a modular version of the SPS has been developed to increase accuracy of the hip reconstruction [34] regarding the leg length and offset.

The SPS stem may tend to increase femoral anteversion consequently increasing the risk of anterior dislocation. Therefore, based on previously reported anatomic studies [33, 34], the extracanalar part of the SPS stem will be modified soon (SPS Evolution®). Our data confirm the medium-term durability of fixation with this implant. However, a longer followup is required to confirm continued durability and maintenance of function.
